# RhoA/ROCK-1 Signaling Pathway and Oxidative Stress in Coronary Artery Disease Patients

**DOI:** 10.21470/1678-9741-2020-0525

**Published:** 2022

**Authors:** Eda Dokumacioglu, Ibrahim Duzcan, Hatice Iskender, Arzu Sahin

**Affiliations:** 1 Department of Nutrition and Dietetics, Faculty of Healthy Sciences, Artvin Çoruh University, Artvin, Turkey.; 2 Department of Cardiovascular Surgery, SBU Kanuni Training and Research Hospital, Trabzon, Turkey.; 3 Department of Physiology, Faculty of Medicine, Uşak University, Uşak, Turkey.

**Keywords:** Coronary Artery Disease, Malondialdehyde, RHOA protein, human, Rho-Associated Kinases, Oxidative Stress, Superoxide Dismutase, Signal Transduction

## Abstract

**Introduction:**

Coronary artery disease (CAD) is an ischemic condition that occurs as a result of partial or complete interruption of blood flow by narrowing or complete blockage of the vessels supplying the heart, which are called coronary arteries. Our objective in this study is to investigate the RhoA/Rho-associated kinase (ROCK)-1 signaling pathway and oxidative stress in CAD patients.

**Methods:**

A total of 81 individuals aged between 40-70 years - including 45 patients (15 females and 30 males) who were admitted to the Artvin State Hospital Cardiovascular Surgery Clinic and were diagnosed with CAD and 36 healthy volunteers (15 females and 21 males) - participated in this study. Serum samples were tested for total cholesterol, triglyceride, low-density lipoprotein, high-density lipoprotein, malondialdehyde (MDA), superoxide dismutase (SOD), RhoA, and ROCK-1 values.

**Results:**

Serum RhoA, MDA levels, and ROCK-1 activity in the CAD group were found to be statistically significantly higher than in the control group (*P*<0.001). Concordantly, serum SOD activity was found to be statistically significantly lower in the CAD group than in the control group (*P*<0.001).

**Conclusion:**

Inhibition of the activity of RhoA/ROCK-1 pathway would be beneficial in treating cardiovascular diseases since this pathway plays an important role in the development of these diseases.

**Table:** 

Abbreviations, acronyms & symbols	
CAD	= Coronary artery disease
DNA	= Deoxyribonucleic acid
ELISA	= Enzyme-linked immunosorbent assay
HDL	= High-density lipoprotein
LDL	= Low-density lipoprotein
MDA	= Malondialdehyde
NBT	= Nitroblue tetrazolium
PUFAs	= Polyunsaturated fatty acids
ROCK	= Rho-associated kinase
ROS	= Reactive oxygen species
SOD	= Superoxide dismutase
TC	= Total cholesterol
TG	= Triglyceride
VSMCs	= Vascular smooth muscle cells

## INTRODUCTION

Coronary artery disease (CAD) is an ischemic condition that occurs as a result of partial or complete interruption of blood flow by narrowing or complete blockage of the vessels supplying the heart, which are called coronary arteries^[1]^. As the leading cause of deaths, CAD is responsible for approximately 48% of deaths in the world population. About 17.3 million people died due to heart attack in 2008, and the World Health Organization predicted that around 23.6 million people will die from cardiovascular disease by 2030^[2]^. Although atherosclerosis is the primary factor causing CAD, many other reasons such as coronary vasospasm, coronary artery embolism, coronary artery anomalies, vasculitis, rheumatic diseases, and trauma can also lead to CAD^[3]^. Atherosclerosis is a chronic inflammatory disease characterized by the formation of fibrous-fatty plaques called “atheroma” in the intimal layers of the medium and large arteries^[4]^.

Epidemiological studies have clearly determined today the relationship between the risk of developing CAD and total cholesterol (TC), triglyceride (TG), low-density lipoprotein (LDL), and high-density lipoprotein (HDL) levels. HDL level was shown to be inversely associated with the risk of developing CAD, because cholesterol is carried mostly by HDL. Therefore, an increase in HDL level is correlated with a decrease in CAD risk. Thus, a close relationship was determined between HDL metabolism, reverse cholesterol transport, and atherogenesis, and the course of CAD can change according to how these three different processes function. The inverse relationship between HDL and the formation of atherosclerosis is partially due to factors other than cholesterol transport^[5]^.

RhoA is a small, guanosine-5’-triphosphate binding protein, bound to the plasma membrane. It is known that Rho regulates the Ca^+2^ sensitivity of vascular smooth muscle cells (VSMCs) by inhibiting myosin phosphatase activity^[6]^. The enzyme called Rho-associated kinase (ROCK) is one of the key targets of Rho proteins in the regulation of cytoskeletal changes. ROCKs are the principal mediators of RhoA activity. ROCK is a serine/threonine protein kinase with a molecular weight of about 160 kDa. There are two isoforms of the Rho-kinase enzyme, ROCK-1 and ROCK-2. Both enzymes are expressed in all cells^[7]^. The RhoA/ROCK signaling pathway is shown to have important functions on vascular physiology and cardiovascular disorders. Although it is recognized as the major regulator of cell contraction, the ROCK enzyme is also known to control migration, proliferation, cell apoptosis, and gene transcription and differentiation. Therefore, ROCK activation appears to be a key factor in initiating the angiogenic process through increased endothelial permeability and migration^[8,9]^. The RhoA/ROCK-1 signaling pathway is known to be associated with several pathological conditions including hypertension, atherosclerosis, stroke ischemia-reperfusion injury, and heart failure^[10,11]^.

Oxidative stress has an important role in the initiation and progression of CAD. And oxidative stress occurs as a result of excessive free oxygen radical production and inadequate antioxidant defense against oxidant radicals^[12]^. Oxidative and antioxidative parameters play an important role in the pathophysiology of atherosclerosis and atherosclerosis-induced CAD. Malondialdehyde (MDA) is one of the important parameters of lipid peroxidation. Oxidative stress increases in correlation with the increase in MDA levels in the development of CAD^[13]^. Oxidative stress can increase reactive oxygen species (ROS) reducing the formation of antioxidant defenses. The reduction of activity of antioxidant enzymes such as superoxide dismutase (SOD) facilitates the oxidative aggression to the cells, especially in subjects with CAD^[14]^. Some authors have demonstrated that in the early stages of CAD, SOD level increased to protect and prevent lipid peroxidation whereas they decreased significantly with the worsening of the disease^[15]^.

The RhoA/ROCK-1 pathway plays an important role in various cellular events involved in the pathogenesis of cardiovascular diseases as well as in the development of the effects of many vasoactive substances. Our objective in this study is to investigate the RhoA/ROCK-1 signaling pathway and oxidative stress in CAD patients.

## METHODS

Ethical approval for the study was obtained from the Ethics Committee of Non-Invasive Clinical Research, Faculty of Medicine of Uşak University, in the session with date 12.12.2018 and number 2018/16, and written consent was obtained from the provincial health directorate of Artvin. The study was conducted in accordance with the guidelines proposed in the Declaration of Helsinki and was approved by the local ethics committee. Written informed consent was obtained from all patients. A total of 81 individuals aged between 40-70 years - including 45 patients (15 females and 30 males) who were admitted to the Artvin State Hospital Cardiovascular Surgery Clinic and were diagnosed with CAD and 36 healthy volunteers (15 females and 21 males) - participated in this study. The volunteers were questioned about atherosclerosis risk factors such as hypertension, diabetes, heart disease, family history of early myocardial infarction, and smoking, and those who meet these criteria were eliminated; ultimately, 36 individuals were included. However, chronic renal failure, chronic liver disease, thyroid diseases, an active infection, cancer history, and chronic inflammatory disease history were determined as exclusion criteria for both groups. All the participants were non-smokers and non-drinkers.

### Blood Sample Collection and Storage

Venous blood samples were taken after 10-12 hours of fasting into tubes with no anticoagulants for biochemical tests. These samples were centrifuged at 4000 rpm for 10 minutes after coagulation was completed to obtain serum samples. After centrifugation, serum samples were tested for TC, TG, LDL, and HDL values. After the required immediate routine tests, the remaining serum samples were stored at -80 ºC until testing for MDA, SOD, RhoA, and ROCK-1.

### Test Protocol of RhoA and ROCK-1 Enzyme-linked Immunosorbent Assay (ELISA) Kits

RhoA and ROCK levels of the study groups were measured by the ELISA method, using ELISA test kits (MyBioSource, Inc., San Diego, California, United States of America). The ELISA method is based on investigating activity of the enzyme linked to the antibody, on the basis of antigen-antibody relationship. The principle is based on showing the antigen-antibody complex formed as a result of the reaction between the antigen or antibody labeled and the enzyme and free antigen or antibody, in the presence of an enzyme-specific substrate. RhoA results are presented in pg/ml. ROCK-1 results are presented in ng/ml.

### Measurement of Superoxide Dismutase Enzyme Activity

SOD enzyme activity was measured using the method developed by Sun et al.^[16]^ In the measurement of serum SOD activity, a color is formed when the superoxide radical produced by the xanthine-xanthine oxidase system reduces the nitroblue tetrazolium (NBT). The resulting color change, that is the reduction of NBT by superoxide radical, ends with the formation of blue colored formazan, giving the maximal absorbance, and the change is determined by spectrophotometer at 560 nm. The results are presented in U/ml.

Measurement of Malondialdehyde Levels

MDA levels were measured using the method developed by Ohkawa et al.^[17]^ The principle of the test is based on measuring the intensity of the pink-red color spectrophotometrically at 532 nm wavelength; where the color was displayed by the compound formed by MDA with thiobarbituric acid in hot environment, and MDA is one of the end products produced in the peroxidation of polyunsaturated fatty acids (PUFAs). The results are presented in nmol/ml.

### Statistical Analysis

Statistical analyses were performed using the IBM Corp. Released 2013, IBM SPSS Statistics for Windows, Version 22.0, Armonk, NY: IBM Corp. Numerical data were given as mean ± standard deviation. Kolmogorov-Smirnov test was used for normal distribution of data. One-way analysis of variance and independent sample *t*-test were used for comparing more than two variables. For intragroup significance, Tukey’s least significance difference test was used for variables with homogenous variances, and Mann-Whitney U test for non-homogeneous variables. For the correlation analysis of the data, Pearson’s correlation analysis was performed for the data with normal distribution, and Spearman’s correlation analysis for the data with non-normal distribution. *P*<0.05 was determined as the threshold for statistical significance.

## RESULTS

Forty-five patients with CAD were included in our study (mean age 58.07± 6.51 years; 30 males and 15 females). Thirty-six healthy volunteers were included in the control group (mean age 56,75± 8,27 years; 21 males and 15 females).

**Fig. 1:**
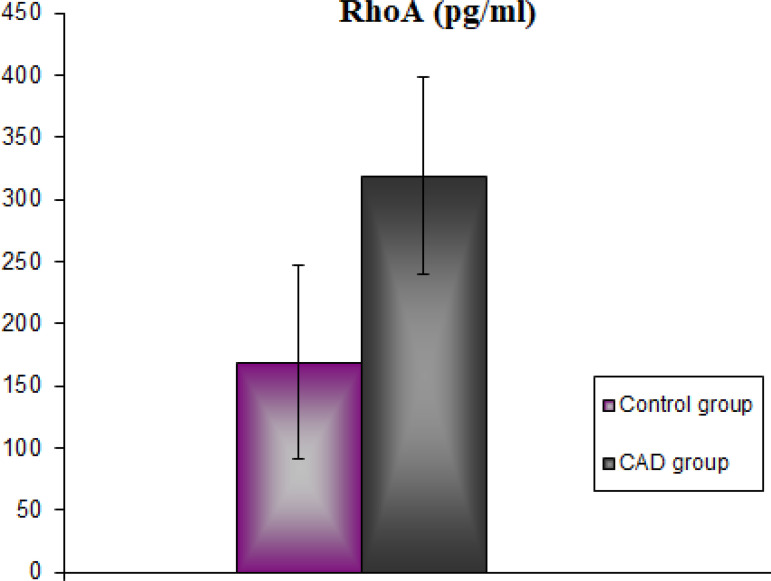
Serum RhoA levels of the groups. CAD=coronary artery disease.

**Fig. 2:**
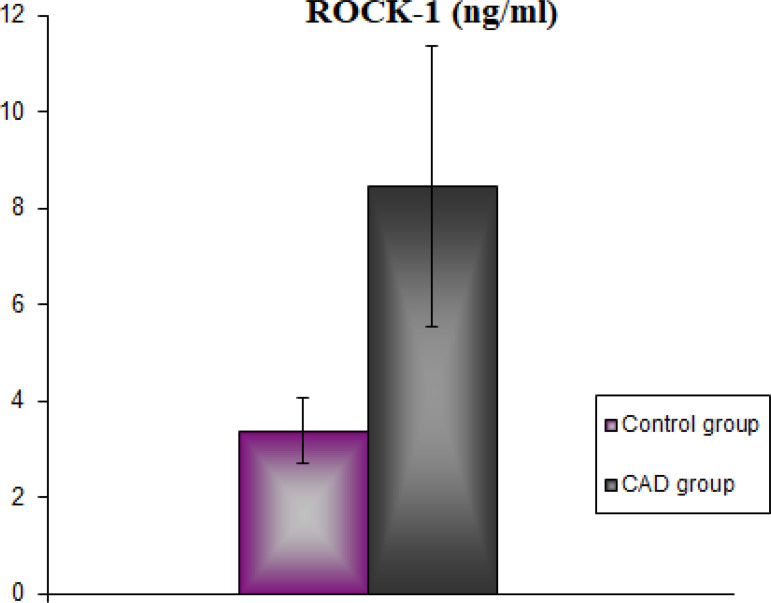
Serum Rho-associated kinase (ROCK)-1 activity of the groups. CAD=coronary artery disease

**Fig. 3:**
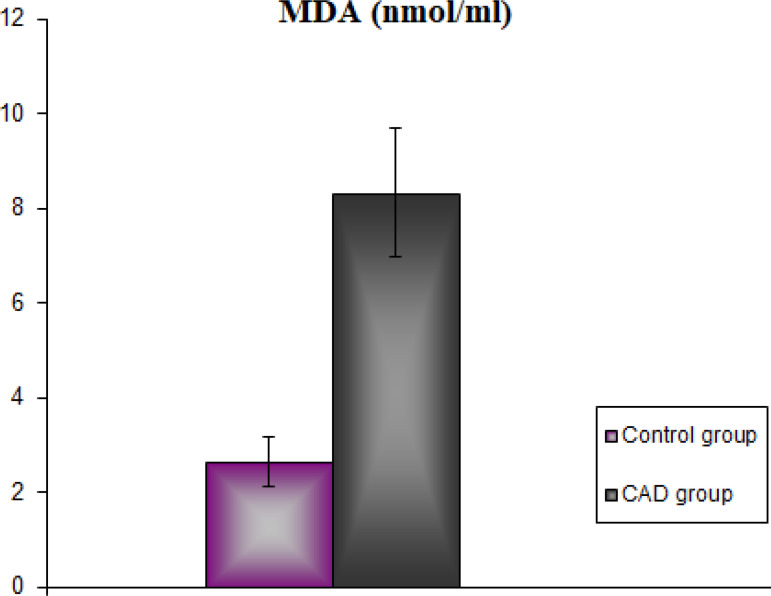
Serum malondialdehyde (MDA) levels of the groups. CAD=coronary artery disease.

**Fig. 4:**
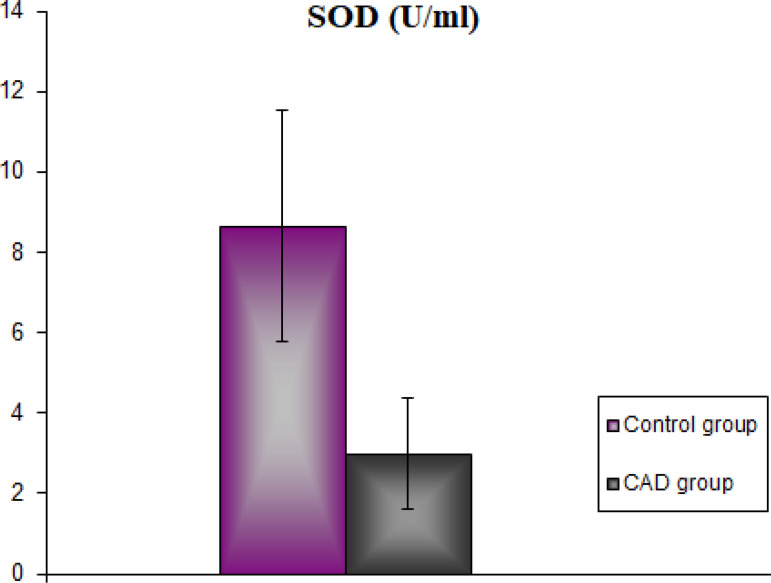
Serum superoxide dismutase (SOD) activity of the groups. CAD=coronary artery disease.

The demographic and clinical characteristics of the CAD group and the healthy control group are shown in Table 1. There was no statistical difference between the groups in terms of age and body weight (*P*>0.05). TC, TG, and LDL levels were found to be statistically significantly higher in the CAD group than in the control group (*P*<0.001). However, serum HDL levels were statistically significantly lower in the CAD group than in the control group (*P*<0.001). In our study, serum RhoA levels in the CAD group were found to be statistically significantly higher than in the control group (*P*<0.001) (Figure 1). Concordantly, serum ROCK activity was found to be statistically significantly higher in the CAD group than in the control group (*P*<0.001) (Figure 2). Serum MDA levels are shown in Figure 3. According to these findings, MDA levels were found to be statistically significantly higher in the CAD group than in the control group (*P*<0.001). Serum activity of SOD, one of the antioxidant enzymes, is shown in Figure 4. Serum SOD activity was found to be statistically significantly lower in the CAD group than in the control group (*P*<0.001).

**Table 1:** Demographic and clinical characteristics of the groups.

	Control group (n=36)	CAD group (n=45)	*P*-value
**Age (years)**	56.75± 8.27	58.07± 6.51	0.539
**Weight (kg)**	76.26± 8.21	82.45± 6;32	0.561
**TG (mg/dl)**	134.14± 29.82	298.69± 152.74	0.000
**TC (mg/dl)**	134.28± 36.23	236.24± 41.35	0.000
**LDL (mg/dl)**	113.89± 30.65	159.07± 20.25	0.000
**HDL (mg/dl)**	59.25±17.53	36.20 & #x00b1; 7.97	0.000

CAD=coronary artery disease; HDL=high-density lipoprotein; LDL=low-density lipoprotein; TC=total cholesterol; TG=triglyceride

Correlation analysis results of our study data are presented in Table 2. Statistical evaluation of our data displayed a significant positive correlation between RhoA and ROCK-1 (r=0.550, *P*<0.001) and between RhoA and MDA (r=0.587, *P*<0.001), and a significant negative correlation between RhoA and SOD (r=-0.575, *P*<0.001). A significant positive correlation was observed between ROCK-1 and MDA (r=0.728, P<0.001) and between ROCK-1 and LDL (r=0.603, *P*<0.001); and a significant negative correlation (r=-0.717, *P*<0.001) was observed between ROCK-1 and SOD.

**Table 2:** Correlation between biochemical parameters.

		RhoA	ROCK-1	MDA	SOD	TC	TG	LDL
**RhoA**	r-value		0.550	0.587	-575	0,542	0,569	0,507
	*P*-value		< 0.001	< 0.001	< 0.001	< 0.001	< 0.001	< 0.001
**ROCK-1**	r-value	0.550		0.728	-717	0,596	0,665	0,603
	*P*-value	< 0.001		< 0.001	< 0.001	< 0.001	< 0.001	< 0.001
**MDA**	r-value	0.587	0.728		-0,656	0,736	0,721	0,524
	*P*-value	< 0.001	< 0.001		< 0.001	< 0.001	< 0.001	< 0.001
**SOD**	r-value	-575	-717	-0,656		-0,587	-0,658	0,514
	*P*-value	< 0.001	< 0.001	< 0.001		< 0.001	< 0.001	< 0.001

HDL=high-density lipoprotein; LDL=low-density lipoprotein; MDA=malondialdehyde; ROCK=Rho-associated kinase; SOD=superoxide dismutase; TC=total cholesterol; TG=triglyceride

A significant positive correlation was found between MDA and TG (r=0.721, *P*<0.001) and between MDA and LDL (r=0.524, *P*<0.001). A significant negative correlation was observed between MDA and SOD (r=-0.656, *P*<0.001) and between MDA and HDL (r=-0.608, *P*<0.001).

SOD and HDL displayed a significant positive correlation (r=0.538, *P*<0.001). And finally, a significant negative correlation was determined between SOD and TG (r=-0.658, *P*<0.001) and between SOD and LDL (r=-0.514, *P*<0.001).

## DISCUSSION

CAD is the foremost cause of morbidity and mortality in the world. Developing new screening tests for CAD gained acceleration recently because CAD is the first cause of death in developed societies, and many patients present with a severe clinical presentation without showing early symptoms. In the context of new signaling pathways and tests, it is aimed to determine high-risk patients before symptoms appear to prevent major cardiac events. In our study aiming to reveal the relationship between MDA and SOD levels, which are used for evaluating the oxidative stress status of the organism in CAD patients as well as the RhoA/ROCK-1 signal pathway, it was observed that RhoA, ROCK-1, and MDA levels of the CAD group increased compared to the control group, and SOD levels decreased.

In recent years, the RhoA/ROCK-1 signaling pathway aroused interest among researchers of cardiovascular diseases. The first reason for this interest is that the RhoA/ROCK signaling pathway plays an important role in various cellular functions involved in the pathogenesis of cardiovascular diseases. Another reason is the considerable influence of RhoA/ROCK pathway over various vasoactive substances involved in the pathogenesis of cardiovascular diseases, such as angiotensin II, 5-hydroxytryptamine, thrombin, and platelet-derived growth factor. Nonetheless, statins, which are 3-hydroxy-3-methylglutaryl coenzyme A (or HMG-CoA) reductase inhibitors, were also reported to have inhibitory effects on the RhoA/ROCK signaling pathway^[18,19]^. Hartman et al.^[20]^ reported that increased ROCK enzyme activities in the processes leading to cardiovascular diseases such as hypertension, angina pectoris, heart failure, and stroke play an important role in the pathogenesis of these diseases. At the same time, they also stated that pharmacological inhibition of ROCK signaling provided considerable improvements in the medical conditions of cardiovascular patients. In our CAD group, a significant increase was determined in both RhoA levels and ROCK-1 activity. Our findings are compatible with the literature. Aghajanian et al.^[21]^ reported in their study that superoxide radicals cause an increase in RhoA levels. In another study by Knock et al.^[22]^, superoxide radicals were also noted to cause an increase in ROCK activities.

Free radicals are molecules containing unpaired electrons generated through biochemical redox reactions that occur during cell metabolism. It is well known that oxidative stress and free radicals have important roles in the process of developing cardiovascular diseases^[23]^. MDA, a carbonyl group produced during lipid peroxidation, is widely used in determining oxidative stress. Increased MDA levels in CAD were demonstrated in several clinical studies^[24,25]^. It was reported in the literature that serum MDA level was a diagnostic parameter in patients with CAD and that there was a strong relation between MDA levels, LDL oxidation, and the development of coronary lesions^[26]^.

In our study, significantly increased MDA and LDL levels and decreased SOD levels were also observed in the CAD group. Our findings are compatible with the results in the literature. A strong positive correlation was found between MDA and LDL whereas a negative correlation was found between MDA and SOD. In addition, an increase in MDA levels showed a positive correlation with RhoA and ROCK levels. It was reported in the literature that excessive ROS production can cause deoxyribonucleic acid (DNA) damage and DNA strand breaks. However, studies indicated that the progression of atherosclerotic plaques were induced by oxidative stress, increasing DNA breaks^[27,28]^. The accumulation of damaged DNA can trigger cell apoptosis or cause permanent cell cycle arrest leading to vascular cell senescence. Vascular cell senescence promotes impaired VSMC proliferation^[29]^.

Increased production of free radicals is counterbalanced by endogenous antioxidants. SOD is one of the most potent enzymatic antioxidants inhibiting free radical formation. Increased SOD levels play a protective role against acute or chronic oxidative damage, including atherosclerosis. But, this increase in SOD levels is provided by foods containing exogenous antioxidants^[30]^. In their study on CAD patients, Gupta et al.^[31]^ reported decreased SOD activities. In our study, SOD levels were also decreased in CAD patients compared to the healthy control group.

When our study groups were examined in terms of dyslipidemia, there was a significant increase in serum TG, TC, and LDL levels in the CAD group, and a decrease in HDL levels was also found. HDL is involved in the reverse cholesterol transport from tissues. It also has antioxidant, anti-inflammatory, and antiapoptotic effects. By virtue of these properties, HDL contributes to the prevention of atherosclerosis by reducing the adverse effects of oxidative stress involved in the initiation and development of this disease^[32]^.

LDL accounts for approximately 70% of plasma cholesterol, providing cholesterol to peripheral tissues. The PUFAs contained in LDL are vulnerable to free radical attacks. Against this oxidative attack, LDL tries to prevent the oxidation of PUFAs by means of vitamin E, which is a powerful antioxidant that LDL contains^[33]^. Increased levels of excessive free radical activity in this region due to decreased antioxidant levels or impaired endothelial function will make PUFAs vulnerable for free radical attacks and initiate a peroxidation chain reaction on LDL. In this case, LDL is changed by modification mechanisms such as glycosylation, oxidation, and acetylation, acquiring atherogenic properties^[34,35]^. Dyslipidemia in our CAD group may have triggered the formation of oxidative stress by causing an increase in MDA levels and a decrease in SOD levels.

Oxidative stress is an important factor in the initiation and progression of CAD, and increased free radicals due to oxidative stress may cause the activation of the RhoA/ROCK signaling pathway.

## CONCLUSION

Our findings suggest that the RhoA/ROCK signaling pathway is important in the pathogenesis of CAD, so the inhibition of the activity of RhoA/ROCK-1 pathway would be beneficial in treating cardiovascular diseases.

**Table:** 

Authors' roles & responsibilities	
ED	Substantial contributions to the conception of the work; drafting the work; agreement to be accountable for all aspects of the work in ensuring that questions related to the accuracy or integrity of any part of the work are appropriately investigated and resolved; final approval of the version to be published
ID	Substantial contributions to the conception of the work; drafting the work; agreement to be accountable for all aspects of the work in ensuring that questions related to the accuracy or integrity of any part of the work are appropriately investigated and resolved; final approval of the version to be published
HI	Substantial contributions to the conception of the work; drafting the work; agreement to be accountable for all aspects of the work in ensuring that questions related to the accuracy or integrity of any part of the work are appropriately investigated and resolved; final approval of the version to be published
AS	Substantial contributions to the conception of the work; drafting the work; agreement to be accountable for all aspects of the work in ensuring that questions related to the accuracy or integrity of any part of the work are appropriately investigated and resolved; final approval of the version to be published
